# Predictors and Outcomes of 30‐Day Readmissions Following Electrical Cardioversion for Atrial Fibrillation: Insights From the Nationwide Readmissions Database

**DOI:** 10.1002/joa3.70406

**Published:** 2026-06-30

**Authors:** Adeena Jamil, Muhammad Umer Sohail, Asad Ali Ahmed Cheema, Syed Usama Ashraf, Anushah Faheem Ilyas, Faizan Abbas, Shaikh Jehanzaib Saeed, Mohammad Rayyan Faisal, Ahmad Murtaza Anwar, Shanzey Rai, Syeda Unzila Tirmizi, Muhammad Uzair, Tooba Shahzad

**Affiliations:** ^1^ Department of Medicine Dow International Medical College, Dow University of Health Sciences Karachi Pakistan; ^2^ Dow University of Health Sciences Karachi Pakistan; ^3^ International School of Medicine, International University of Kyrgyzstan Bishkek Kyrgyzstan; ^4^ Dow International Medical College Karachi Pakistan; ^5^ Department of Medicine Karachi Medical & Dental College/KMU Karachi Pakistan; ^6^ Department of Medicine Fatima Memorial Hospital Lahore Pakistan; ^7^ Aga Khan University Hospital Karachi Pakistan; ^8^ Department of Medicine Mohtarma Benazir Bhutto Shaheed Medical College Mirpur Pakistan; ^9^ Islamic International Medical College Rawalpindi Pakistan; ^10^ Shaheed Mohtarma Benazir Bhutto Medical College Karachi Pakistan; ^11^ Ziauddin Medical College Karachi Pakistan; ^12^ Sargodha Medical College Sargodha Pakistan

**Keywords:** atrial fibrillation, electrical cardioversion, hospital readmission, NRD database, predictors

## Abstract

**Background:**

Atrial Fibrillation (AF) affects 10.5 million adults in the US and is projected to reach 12.1 million by 2030, representing a growing burden. We evaluated predictors of 30‐day readmission after electrical cardioversion (EC) for AF.

**Methods:**

In this retrospective observational cohort study using the Nationwide Readmissions Database (2016–2017), we identified adults with AF undergoing EC. Outcomes evaluated were 30‐day all‐cause readmission, in‐hospital mortality, length of stay (LOS) and inflation‐adjusted hospitalization charges. Multivariable logistic regression identified predictors of 30‐day readmission.

**Results:**

Among 134 114 AF hospitalizations managed with EC, 13 260 (9.9%) were readmitted within 30 days. Readmitted patients (mean age 70, 51% male) had longer index stays (4 vs. 3 days), higher charges ($31 576 vs. $26 896), and more frequent nonhome discharges (11% vs. 6.4%; all *p* < 0.001). Readmissions added $27 994 in costs. In‐hospital mortality was higher during readmission (2.9% vs. 0.7%). Independent predictors included female sex, Medicaid or Medicare coverage, and chronic pulmonary disease, hypertension, diabetes and heart failure (HF) (*p* < 0.001). Higher median household income was associated with lower odds of readmission (*p* < 0.001).

**Conclusion:**

Nearly 1 in 10 AF patients treated with EC was readmitted within 30 days. Female sex, comorbidity burden and lower socioeconomic status were key predictors. Improved post‐discharge care addressing comorbidities alongside rhythm management may reduce early readmissions.

## Graphical abstract

Among 134 114 atrial fibrillation hospitalizations treated with electrical cardioversion in the 2016–2017 NRD, 9.9% resulted in 30‐day readmission. Readmission was associated with higher mortality, longer stay and greater costs. Predictors included cardiometabolic comorbidities, female sex, lower income, Medicare/Medicaid insurance, nonhome discharge, highlighting clinical and socioeconomic risk. Central illustration: Key predictors and outcomes of 30‐day readmission following electrical cardioversion for atrial fibrillation, based on National Readmission Database (2016–2017).

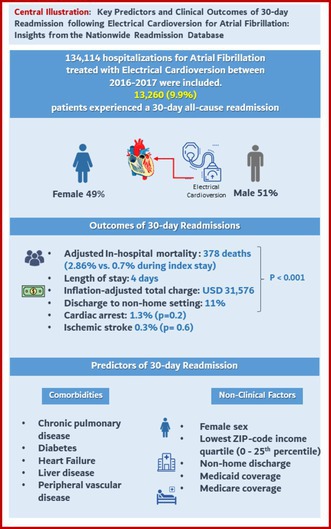

**Abbreviations**
AADs: antiarrhythmic drugs
AF: atrial fibrillation
AHA: American Heart Association
AHRQ: Agency for Healthcare Research and Quality
BMI: body mass index
CA: catheter ablation
CAD: coronary artery disease
CI: confidence interval
CKD: chronic kidney disease
COPD: chronic obstructive pulmonary disease
CPI: Consumer Price Index
DOAC: direct oral anticoagulant
EC: electrical cardioversion
HCUP: Healthcare Cost and Utilization Project
HF: heart failure
ICD‐10‐CM: International Classification of Diseases, 10th Revision, Clinical Modification
ICD‐10‐PCS: International Classification of Diseases, 10th Revision, Procedure Coding System
IHD: ischemic heart disease
IRB: institutional review board
LAAO: left atrial appendage occlusion
LOS: length of stay
NRD: Nationwide Readmissions Database
NSR: normal sinus rhythm
OR: odds ratio
RAAS: renin–angiotensin–aldosterone system
TCM: transitional care management
TIA: transient ischemic attack
US: United States
USD: United States dollars

## 1 Introduction

Atrial fibrillation (AF) is the most prevalent cardiac arrhythmia worldwide. In 2021, AF accounted for an estimated 4.48 million new cases globally, with a total prevalence of 52.55 million and 338 947 deaths [1]. In the United States (US), the burden of AF is rising faster than previously projected. A recent 2024 study reported that at least 10.55 million adults are affected, corresponding to 4.48% of the adult population [2]. This increasing prevalence is driven by an aging population, rising obesity rates, improved screening and diagnostics, and prolonged survival among individuals with AF and other cardiovascular diseases [3]. AF imposes a substantial clinical burden, increasing the risk for stroke, heart failure (HF), and ischemic heart disease (IHD). A large‐scale Nationwide Readmission Database (NRD) analysis reported that 14.4% of patients discharged after an AF hospitalization were readmitted within 30 days, with AF itself being the most common cause of readmission (24.1%). Importantly, the utilization of electrical cardioversion (EC) or catheter ablation (CA) during the first admission was independently associated with a reduced risk of readmission within 30 days [4]. In the USA, the annual economic burden of AF is estimated at $6–26 billion, with nearly 460 000 hospitalizations each year, representing a significant public health challenge [4, 5].

EC is a key nonpharmacological strategy for rhythm restoration in AF, delivering synchronized electrical energy to reestablish normal sinus rhythm (NSR) [6]. The 2023 American Heart Association (AHA) guidelines highlight that rhythm control, including EC, can relieve symptoms, slow disease progression, and reduce hospitalization and mortality, particularly in patients with HF [3]. In clinical practice, EC is frequently used in hospitalized patients with hemodynamic instability or persistent symptoms despite rate control. The utilization of EC has increased significantly, with inpatient use rising from 4.3% between 2000 and 2010 to 16.4% by 2014, reflecting broader adoption of rhythm control strategies [7]. Although EC achieves short‐term success rates exceeding 90% in restoring NSR, long‐term maintenance remains challenging, with fewer than 50% of patients sustaining NSR at 1 year [8].

Despite advancements in AF management, hospital readmission rates remain high [4]. Although prior studies have examined AF‐related readmissions, none have specifically characterized outcomes following EC [4]. To our knowledge, this is the first nationally representative study utilizing an all‐payer administrative database to provide real‐world evidence on the incidence, outcomes, and predictors of 30‐day readmission following EC for AF.

## 2 Methods

### 2.1 Ethics Statement

This study used de‐identified, publicly available data from the NRD database. Therefore, institutional review board (IRB) approval and informed consent were not required.

### 2.2 Data Source

This retrospective observational cohort study utilized data from the NRD from 2016 to 2017. We selected the NRD from 2016 to 2017 to ensure a consistent International Classification of Diseases, Tenth Revision, Clinical Modification/Procedure Coding System (ICD‐10‐CM/PCS) coding framework after ICD‐10‐CM/PCS implementation period and because these were the NRD files available to the investigators at the time of data acquisition and analysis. The NRD, one of the largest publicly available all‐payer databases, is sponsored by the Agency for Healthcare Research and Quality as part of the HCUP and enables nationally representative readmission analyses. It contains discharge data collected over the calendar year from multiple states and allows tracking of individual patients across hospitals within a given state during the same year using de‐identified patient linkage numbers, facilitating the capture of readmissions. The 2016 NRD encompassed discharges from 28 states, representing approximately 57% of all US hospitalizations, while the 2017 NRD included nearly 18 million discharges from 285 hospitals, representing about two‐thirds of the US population.

### 2.3 Study Population

**FIGURE 1:**
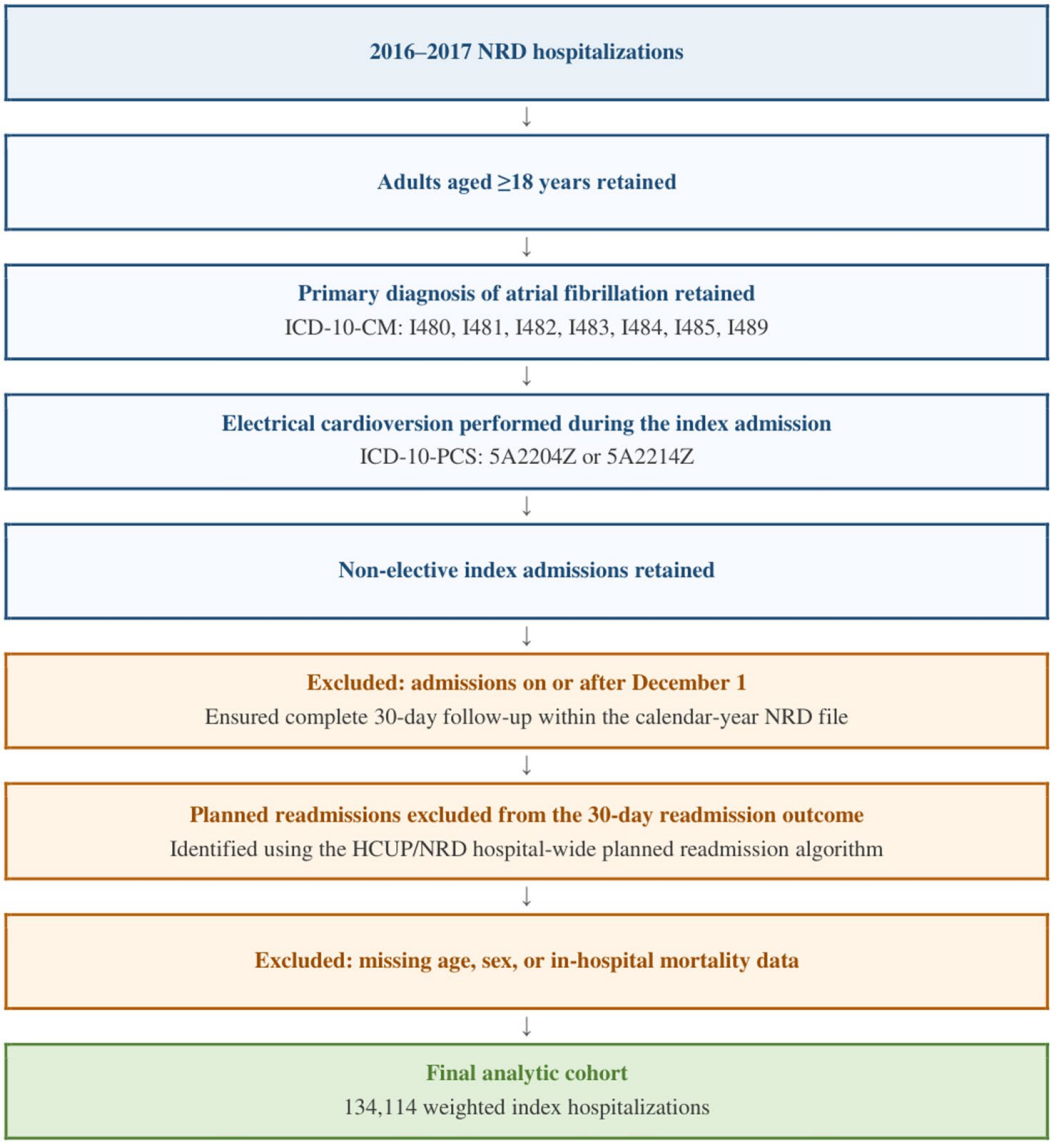
Patient selection flow diagram. Transfer to another short‐term acute care hospital was not applied as an exclusion criterion and was captured only as part of the nonhome discharge disposition variable. No procedure‐specific exclusions were applied. Patients undergoing catheter ablation, thrombectomy, cardiovascular surgery or other in‐hospital procedures were retained if they otherwise met eligibility criteria. Patients who underwent both catheter ablation and electrical cardioversion during the same index admission were included in the analytic cohort if they otherwise met the cohort definition.

The International Classification of Diseases‐10th Revision‐Clinical Modification (ICD‐10‐CM) diagnosis codes I480, I481, I482, I483, I484, I485, and I489 were used to identify hospitalizations with AF. Synchronized EC during the same admission was identified using International Classification of Diseases, Tenth Revision, Procedure Coding System (ICD‐10‐PCS) procedure codes 5A2204Z or 5A2214Z. The index admission was defined as the first hospitalization meeting the inclusion criteria within the study year. To ensure that all patients had a complete 30‐day readmission follow‐up window captured within the same calendar year, we excluded index hospitalizations with admission dates on or after December 1 of each study year, as the NRD is organized as separate calendar‐year files and patient linkage numbers cannot be followed across years. Readmission time was calculated by subtracting the index admission length of stay (LOS) from the number of days between index discharge and the subsequent admission. Only unplanned readmissions occurring within 30 days of discharge were included to reflect acute management of AF. Planned readmissions were identified and excluded using the HCUP/NRD hospital‐wide planned readmission algorithm, which flags non‐acute, scheduled encounters such as staged procedures or elective therapies [9]. Patients younger than 18 years and hospitalizations with missing data on age, sex, or in‐hospital mortality were excluded. Transfer to another short‐term acute care hospital was treated as an index‐hospitalization discharge disposition and was not applied as an exclusion criterion. Therefore, patients discharged or transferred to another acute care facility after the index hospitalization were retained in the analytic cohort if they otherwise met all eligibility criteria. Importantly, no procedure‐specific exclusion was applied for catheter ablation, thrombectomy, cardiovascular surgery, or other in‐hospital procedures. Accordingly, patients who underwent both catheter ablation and electrical cardioversion during the same index admission were included in the analytic cohort if they otherwise met the cohort definition. The stepwise cohort derivation is summarized in Figure 1.

### 2.4 Variables

Demographic variables included age, sex, primary insurance type, and ZIP‐code‐level median household income quartile. Median household income was measured using the HCUP ZIPINC_QRTL variable, an area‐level measure that classifies the estimated median household income of residents in the patient's ZIP Code into national quartiles. Therefore, this variable reflects neighborhood‐level socioeconomic position rather than individual household income and does not account for family size. Income was stratified into quartiles: Quartile 1 (≤ $47 999), Quartile 2 ($48 000–$60 999), Quartile 3 ($61 000–$81 999), and Quartile 4 (≥ $82 000). Hospital‐level variables included bed size, teaching status, and location. Comorbidities were identified using HCUP comorbidity data elements derived from the AHRQ Elixhauser Comorbidity Software for ICD‐10‐CM. In NRD data, the prefix “CM_” denotes HCUP comorbidity measure variables and is used to distinguish these variables from other HCUP data elements. These variables identify pre‐existing conditions based on secondary diagnosis codes and are not intended to represent conditions directly related to the principal reason for hospitalization.

### 2.5 Study Endpoints

The primary endpoint was all‐cause, unplanned hospital readmission within 30 days after discharge from the index AF hospitalization managed with EC. Secondary outcomes included in‐hospital mortality, LOS in days, LOS dichotomized as ≤ 4 versus > 4 days, and total hospitalization charges for the index hospitalization and readmission, with charges inflation‐adjusted to 2017 United States dollars (USD) using Consumer Price Index (CPI) data. We also evaluated nonhome discharge as an index‐hospitalization disposition outcome. Nonhome discharge was defined as any discharge disposition other than home/self‐care, including transfer to another short‐term acute care hospital, discharge to a skilled nursing facility or intermediate care facility, discharge against medical advice, or in‐hospital death. This variable reflected disposition at the end of the index hospitalization only. In contrast, the 30‐day readmission endpoint was defined as a subsequent unplanned hospital admission occurring after discharge from the index hospitalization and within 30 days. Therefore, transfer to another short‐term acute care hospital during the index discharge process was not treated as a readmission event and was not used as an exclusion criterion.

### 2.6 Statistical Analysis

All analyses accounted for the NRD's complex survey design using discharge‐level weights, stratification, and hospital clustering to produce nationally representative estimates. Analyses were performed in R Statistical Language (version 4.5.0; R Foundation for Statistical Computing, Vienna, Austria) using the survey and srvyr packages to incorporate variance estimation for complex surveys. Patient and hospital‐level variables were included in the baseline characteristics for analysis. Baseline characteristics were stratified by 30‐day readmission status using Rao‐Scott adjusted chi‐square tests for categorical variables and design‐based Kruskal–Wallis tests for continuous variables. Missing data were reported using exact counts and percentages and were calculated using the unweighted analytic sample as the denominator. Complete‐case analysis was used for analyses involving variables with missing data. Complete and incomplete cases were compared using standardized mean differences.

Survey‐weighted multivariable logistic regression was used to identify independent predictors of 30‐day readmission, including demographics (age, sex, insurance type, income quartile), comorbidities (Elixhauser conditions and ICD‐10 codes), hospital characteristics (teaching status, bed size, and location), and index hospitalization features (LOS, discharge disposition, and weekend admission). Multicollinearity among model predictors was assessed using generalized variance inflation factors (GVIFs), with GVIF^(1/(2·df)^) used for multi‐level categorical variables. Categorical predictors had pre‐specified reference categories (e.g., female sex, government insurance, and LOS > 4 days). Adjusted odds ratios (ORs) with 95% confidence intervals (CIs) are reported [9]. For readmitted patients, survey‐weighted means, medians, and proportions were summarized for LOS, hospitalization charges, and in‐hospital mortality. We summarized the top 10 causes for readmission with survey‐weighted proportions using the principal diagnosis field, Diagnosis 1 (DX1). A two‐sided *p*‐value < 0.05 was considered statistically significant. This analytic approach is consistent with prior NRD‐based studies that have evaluated outcomes and predictors in AF and other cardiovascular conditions [10, 11]. This study was conducted and reported in accordance with the Strengthening the Reporting of Observational Studies in Epidemiology (STROBE) guidelines [12] and the REporting of studies Conducted using Observational Routinely‐collected health Data (RECORD) extension [13].

## 3 Results

A total of 134 114 weighted index hospitalizations for AF treated with EC were identified from the 2016–2017 NRD. Among these, the 30‐day all‐cause readmission rate was 9.9%, representing an estimated 13 260 patients (Central Illustration). Among 134 114 weighted index hospitalizations, corresponding to 70 338 unweighted index hospitalizations, missing data were confined to two covariates: primary expected payer status in 82 hospitalizations (0.12%) and ZIP‐code‐level median household income quartile in 978 hospitalizations (1.38%). In total, 1052 unique records had incomplete covariate data (1.49%), indicating overlap between missingness in these two variables. Comparison of complete and incomplete records showed no meaningful imbalance in readmission status, in‐hospital mortality, length of stay, total charges, nonhome discharge, comorbidity profile, or key demographic characteristics.

### 3.1 Baseline Characteristics

When stratified by 30‐day readmission status during index admissions, readmitted patients were significantly older (mean age: 70 vs. 68 years) and included a higher proportion of females (49% vs. 42%), although the majority were male. Medicare was the predominant payer overall (63%), with greater coverage among readmitted patients (71% vs. 62%, *p* < 0.001). Readmissions disproportionately affected patients in the lowest income quartile (29% vs. 25%; *p* < 0.001), while patients in the highest quartile were less likely to be readmitted (18% vs. 22%). Hospitals with large‐bed sizes accounted for 60% of readmissions. Comorbidity burden was higher among readmitted patients, including diabetes (35% vs. 27%), HF (60% vs. 49%), chronic pulmonary disease, peripheral vascular disease, prior stroke, and liver disease (Table 1).

**TABLE 1:** Baseline characteristics of patients readmitted following electrical cardioversion admitted for atrial fibrillation.

Characteristic	Overall *N* = 134 114^a^	Without 30‐day readmission *N* = 120 854^a^	With 30‐day readmission *N* = 13 260^a^	*p* ^b^
Age (years)	68 (13)	68 (13)	70 (12)	< 0.001
Sex				
Male	77 234 (58%)	70 483 (58%)	6751 (51%)	< 0.001
Female	56 880 (42%)	50 371 (42%)	6509 (49%)	
Primary expected payer				
Private	35 813 (27%)	33 368 (28%)	2445 (18%)	< 0.001
Medicaid	7567 (5.6%)	6694 (5.5%)	873 (6.6%)	
Medicare	83 877 (63%)	74 527 (62%)	9350 (71%)	
Other	6725 (5.0%)	6147 (5.1%)	578 (4.4%)	
Median household income quartile				
0–25th percentile	33 367 (25%)	29 577 (25%)	3790 (29%)	< 0.001
26th–50th percentile	35 758 (27%)	32 099 (27%)	3659 (28%)	
51st–75th percentile	34 857 (26%)	31 607 (26%)	3250 (25%)	
76th–100th percentile	28 401 (21%)	26 001 (22%)	2400 (18%)	
Admission day				
Monday–Friday	108 246 (81%)	97 611 (81%)	10 636 (80%)	0.3
Saturday–Sunday	25 867 (19%)	23 243 (19%)	2624 (20%)	
Hospital bed size				
Small	18 593 (14%)	16 948 (14%)	1645 (12%)	0.002
Large	77 448 (58%)	69 535 (58%)	7913 (60%)	
Medium	38 073 (28%)	34 371 (28%)	3702 (28%)	
Hospital location and teaching status				
Metropolitan, nonteaching	33 346 (25%)	29 885 (25%)	3460 (26%)	0.13
Metropolitan, teaching	91 203 (68%)	82 352 (68%)	8852 (67%)	
Nonmetropolitan	9565 (7.1%)	8617 (7.1%)	948 (7.1%)	
Comorbidities				
Diabetes	37 329 (28%)	32 696 (27%)	4633 (35%)	< 0.001
Congestive heart failure	67 788 (51%)	59 801 (49%)	7987 (60%)	< 0.001
Alcohol use	7041 (5.2%)	6409 (5.3%)	632 (4.8%)	0.066
Peripheral vascular disease	14 023 (10%)	12 296 (10%)	1727 (13%)	< 0.001
Hypertension	82 880 (62%)	75 140 (62%)	7740 (58%)	< 0.001
Obesity	36 264 (27%)	32 870 (27%)	3393 (26%)	0.009
Previous stroke or transient ischemic attack	11 093 (8.3%)	9803 (8.1%)	1290 (9.7%)	< 0.001
Chronic pulmonary disease	32 328 (24%)	27 836 (23%)	4492 (34%)	< 0.001
Liver disease	3767 (2.8%)	3306 (2.7%)	461 (3.5%)	0.001

*Note:* Missing data were calculated using the unweighted analytic sample denominator of 70 338 index hospitalizations. Missingness was confined to primary expected payer status in 82 hospitalizations (0.12%) and ZIP‐code‐level median household income quartile in 978 hospitalizations (1.38%). Overall, 1052 unique records had incomplete covariate data (1.49%), reflecting overlap between missingness in these two variables. All other covariates in Table 1 had complete data. Complete versus incomplete cases were generally balanced for readmission, mortality, length of stay, total charges, nonhome discharge, and comorbidities, with standardized mean differences < 0.10; small imbalances were observed for age and sex.

^a^ Mean (SD); *n* (%).

^b^ Design‐based Kruskal–Wallis test; Pearson's *X* ^2^: Rao and Scott adjustment.

### 3.2 Outcomes of Index Hospitalization

Patients who experienced 30‐day all‐cause readmissions had significantly different index hospitalization outcomes compared to those who were not readmitted. Readmitted patients had longer median LOS at index hospitalization (4 vs. 3 days; *p* < 0.001), higher inflation‐adjusted total charges ($31 576 vs. $26 896; *p* < 0.001), and were more frequently discharged to nonhome settings (11% vs. 6.4%; *p* < 0.001). No significant differences were observed in the occurrence of cardiac arrest or ischemic stroke between the two groups (Table 2).

**TABLE 2:** Index hospitalization outcomes stratified by 30‐day readmission status among patients with atrial fibrillation undergoing electrical cardioversion.

Characteristic	Overall *N* = 134 114^a^	Without 30‐day readmission *N* = 120 854^a^	With 30‐day readmission *N* = 13 260^a^	*p* ^b^
Index in‐hospital mortality	794 (0.6%)	794 (0.7%)	—	< 0.001
In‐hospital mortality during 30‐day readmission	—	—	378 (2.86%)	—
Length of stay (days)	3.00 (2.00, 5.00)	3.00 (2.00, 5.00)	4.00 (2.00, 6.00)	< 0.001
Inflation‐adjusted total charges ($)	27 319 (16 734, 47 646)	26 896 (16 500, 46 836)	31 576 (19 118, 55 717)	< 0.001
Discharged to nonhome setting	9219 (6.9%)	7788 (6.4%)	1430 (11%)	< 0.001
Cardiac arrest	1454 (1.1%)	1287 (1.1%)	167 (1.3%)	0.2
Ischemic stroke	362 (0.3%)	322 (0.3%)	40 (0.3%)	0.6

*Note:* Among patients with a 30‐day readmission, in‐hospital mortality during the readmission hospitalization was 2.86% (378 deaths; 95% CI: 2.42%–3.29%).

^a^ *n* (%); Median (Q1, Q3).

^b^ Pearson's *X* ^2^: Rao and Scott adjustment; design‐based Kruskal–Wallis test.

### 3.3 Outcomes of Readmission

After adjusting for confounders, multivariable logistic analysis revealed that during readmission hospitalization, patients had a median LOS of 3 days and incurred median inflation‐adjusted charges of $27 994. During readmission, 378 patients died, corresponding to an in‐hospital mortality rate of 2.86% (95% CI: 2.42%–3.29%), markedly higher than the 0.7% mortality observed in the non‐readmitted cohort.

### 3.4 Risk Factors for 30‐Day Readmission

#### 3.4.1 Socio‐Demographic Risk Factors

**FIGURE 2:**
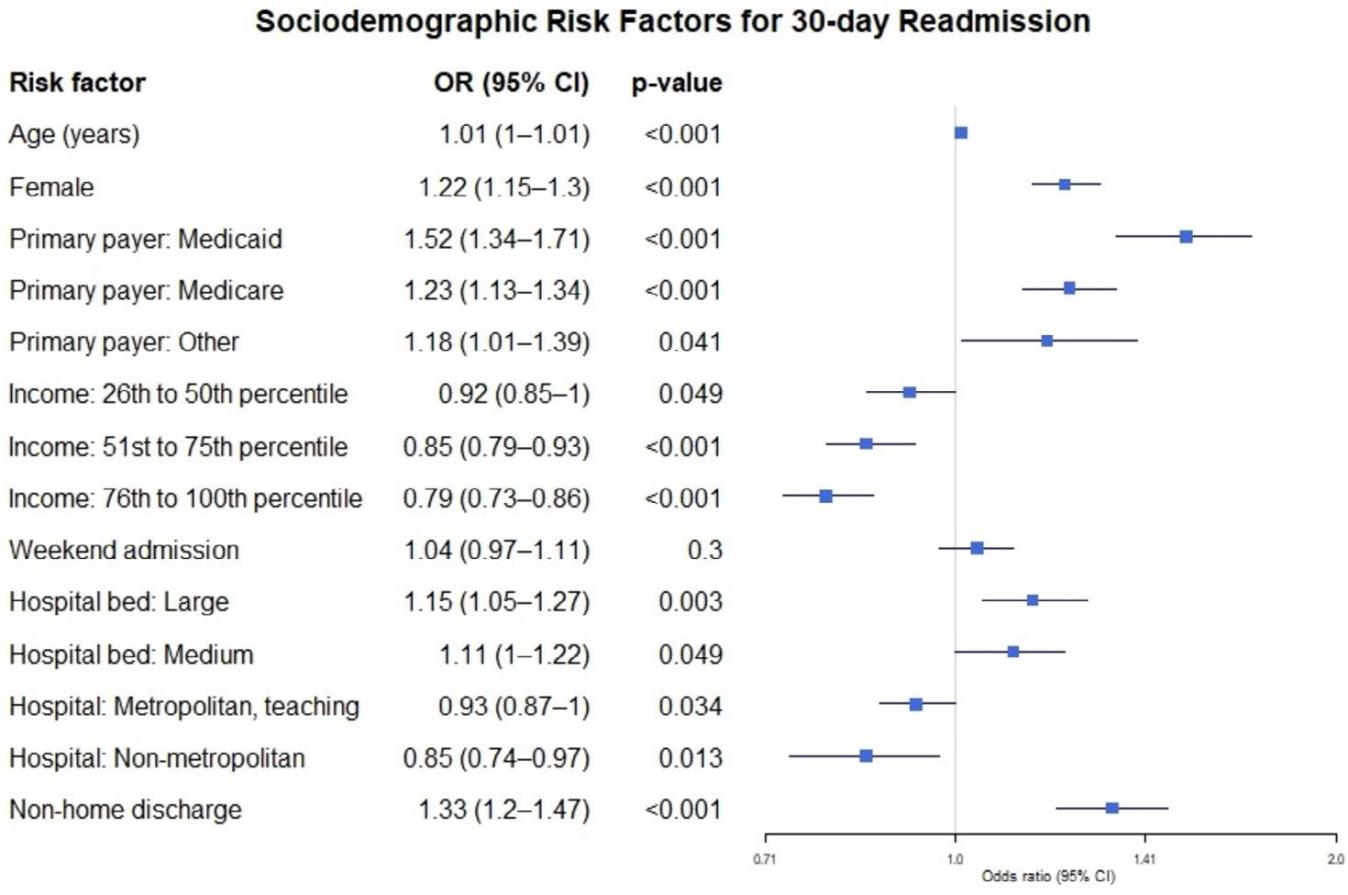
Forest plot showing adjusted odds ratio (95% CI) for sociodemographic risk factors associated with 30‐day readmission after electrical cardioversion for atrial fibrillation.

In adjusted multivariable logistic analysis, several socio‐demographic factors were independently associated with increased odds of 30‐day readmission following EC for AF. Each additional year of age modestly increased risk (OR: 1.01; 1–1.01; *p* < 0.001). Females had higher odds of readmission (OR: 1.22; 1.15–1.30; *p* < 0.001). Compared with private insurance, Medicaid (OR: 1.52; 1.34–1.71; *p* < 0.001) and Medicare coverage (OR: 1.23; 1.13–1.34; *p* < 0.001) were associated with higher risk. Nonhome discharge also increased readmission risk (OR: 1.33; 1.2–1.47; *p* < 0.001). Compared with small‐bed‐size hospitals, both large‐bed‐size hospitals (OR: 1.15; 95% CI: 1.05–1.27; *p* = 0.003) and medium‐bed‐size hospitals (OR: 1.11; 95% CI: 1.00–1.22; *p* = 0.049) were associated with higher odds of 30‐day readmission, whereas teaching and nonmetropolitan facilities were associated with lower odds. By income status, patients in the top 3rd and 4th quartiles had significantly lower odds of readmission (Table 3, Figure 2).

**TABLE 3:** Multivariable analysis of risk factors in atrial fibrillation patients associated with 30‐day readmission following electrical cardioversion.

Characteristic	OR	95% CI	*p*
Age (years)	1.01	1.00, 1.01	< 0.001
Sex			
Male	—	—	
Female	1.22	1.15, 1.30	< 0.001
Primary expected payer			
Private	—	—	
Medicaid	1.52	1.34, 1.71	< 0.001
Medicare	1.23	1.13, 1.34	< 0.001
Other	1.18	1.01, 1.39	0.041
Median household income quartile			
0–25th percentile	—	—	
26th–50th percentile	0.92	0.85, 1.00	0.049
51st–75th percentile	0.85	0.79, 0.93	< 0.001
76th–100th percentile	0.79	0.73, 0.86	< 0.001
Admission day			
Monday–Friday	—	—	
Saturday–Sunday	1.04	0.97, 1.11	0.3
Hospital bed size			
Small	—	—	
Large	1.15	1.05, 1.27	0.003
Medium	1.11	1.00, 1.22	0.049
Hospital location and teaching status			
Metropolitan, nonteaching	—	—	
Metropolitan, teaching	0.93	0.87, 1.0	0.034
Nonmetropolitan	0.85	0.74, 0.97	0.013
Diabetes			
No	—	—	
Yes	1.36	1.28, 1.44	< 0.001
Congestive heart failure			
No	—	—	
Yes	1.34	1.27, 1.42	< 0.001
Alcohol use			
No	—	—	
Yes	1.03	0.90, 1.16	0.7
Peripheral vascular disease			
No	—	—	
Yes	1.14	1.05, 1.24	0.003
Hypertension			
No	—	—	
Yes	0.85	0.80, 0.90	< 0.001
Obesity			
No	—	—	
Yes	0.90	0.84, 0.96	0.002
Previous stroke or transient ischemic attack			
No	—	—	
Yes	1.05	0.96, 1.15	0.3
Chronic pulmonary disease			
No	—	—	
Yes	1.51	1.42, 1.61	< 0.001
Liver disease			
No	—	—	
Yes	1.22	1.04, 1.43	0.016
Cardiogenic shock			
No	—	—	
Yes	0.88	0.67, 1.16	0.4
Cardiac arrest			
No	—	—	
Yes	0.46	0.30, 0.71	< 0.001
Ischemic stroke			
No	—	—	
Yes	0.94	0.59, 1.50	0.8
Nonhome discharge			
No	—	—	
Yes	1.33	1.20, 1.47	< 0.001

Abbreviations: CI, confidence interval; OR, odds ratio.

#### 3.4.2 Comorbidities Associated With 30‐Day Readmission

**FIGURE 3:**
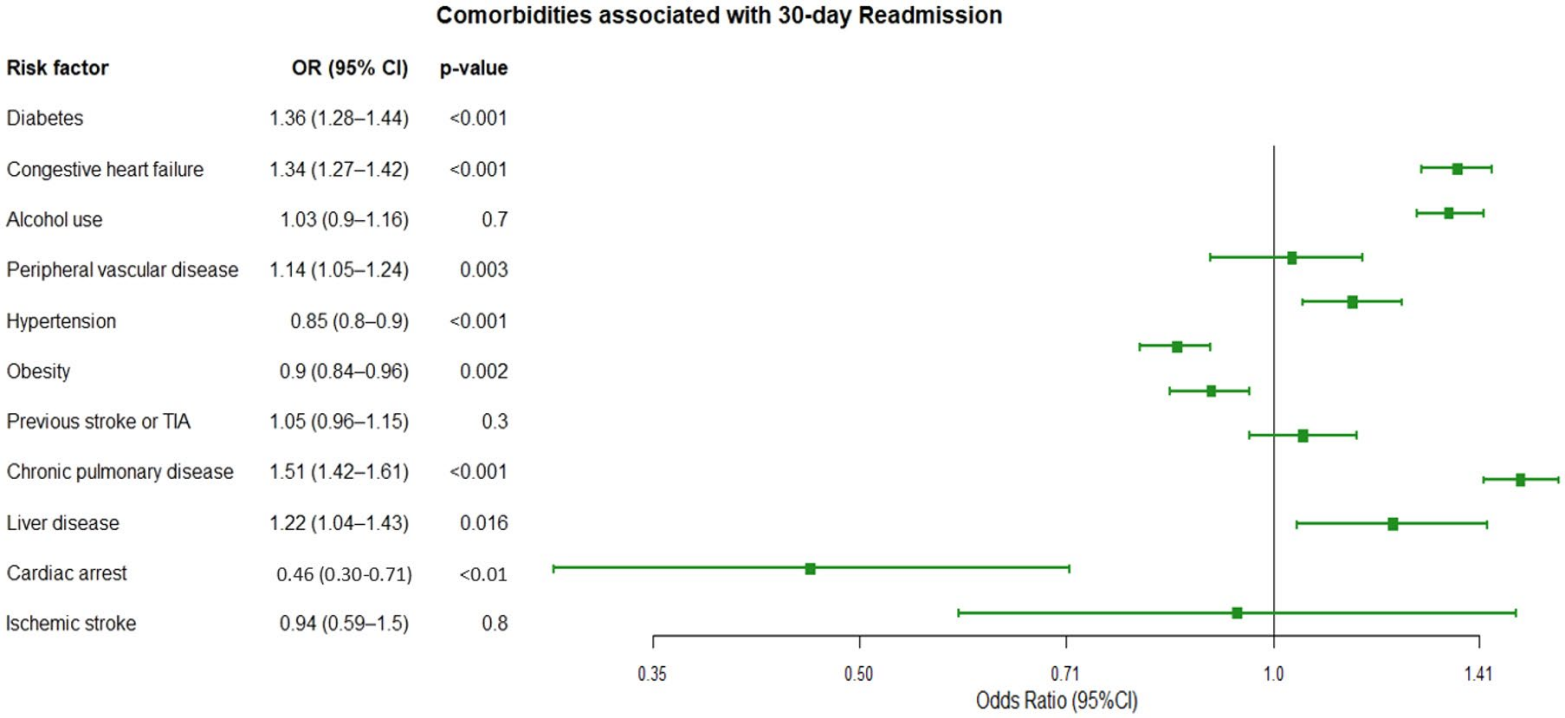
Forest plot showing adjusted odds ratio (95% CI) for comorbidities associated with 30‐day readmission after electrical cardioversion for atrial fibrillation.

The strongest comorbid predictors of readmission were chronic obstructive pulmonary disease (COPD) (OR: 1.51; 1.42–1.61; *p* < 0.001), diabetes (OR: 1.36; 1.28–1.44; *p* < 0.001), HF (OR: 1.34; 1.27–1.42; *p* < 0.001), liver disease, and peripheral vascular disease. Hypertension and obesity were associated with reduced readmission rates (Table 3, Figure 3). No evidence of meaningful multicollinearity was observed among model predictors; the maximum GVIF^(1/(2·df)^) was 1.41.

#### 3.4.3 Principal Diagnosis for 30‐Day Readmission

Among readmitted patients, AF and atrial flutter arrhythmia were the most common principal causes for 30‐day readmission, accounting for 32.31% and 5.29% of readmissions, respectively. Heart failure–related diagnoses accounted for an additional 13.3%. A complete list of the top 10 principal diagnoses is provided in Table 4.

**TABLE 4:** Top 10 principal diagnosis codes for 30‐day readmission.

ICD‐10‐CM code	Principal diagnosis (DX1)	Proportion of readmissions (%)
I48.0	Paroxysmal atrial fibrillation	16.50
I48.91	Unspecified atrial fibrillation	8.35
I48.1	Persistent atrial fibrillation	7.46
I48.92	Unspecified atrial flutter	5.29
I11.0	Hypertensive heart disease with heart failure	4.40
I13.0	Hypertensive heart and chronic kidney disease with heart failure	4.08
A41.9	Sepsis, unspecified organism	2.93
I50.33	Acute on chronic diastolic heart failure	2.50
I50.23	Acute on chronic systolic heart failure	2.33
I49.5	Sick sinus syndrome	1.88

*Note:* Values are survey‐weighted proportions among readmissions and reflect the principal diagnosis field (DX1).

Abbreviations: DX1, principal diagnosis; ICD‐10‐CM, International classification of diseases, tenth revision, clinical modification.

## 4 Discussion

In this study of 134 114 weighted hospitalizations for AF treated with EC during 2016 and 2017, nearly one in 10 patients experienced a 30‐day all‐cause readmission. Readmitted patients were older and had a greater comorbidity burden, including DM, COPD, HF, and peripheral vascular disease. Despite a nearly equal sex distribution in the readmitted cohort, female sex emerged as an independent predictor of 30‐day readmission in our multivariable model. In addition, readmitted patients had more extended index hospital stays, higher healthcare costs, and were more frequently discharged to nonhome settings at index hospitalization. Mortality was significantly higher during readmission compared with the index hospitalization. Multivariable analysis identified older age, female sex, public insurance, lower income, and comorbidities such as COPD, DM, HF, liver disease, and peripheral vascular disease as key predictors of readmission.

The 30‐day readmission rates among patients receiving EC during the initial hospitalization were approximately one in 10 in our study, which is lower than those reported in previous analyses of AF readmissions. Compared with general AF hospitalization cohorts, our EC cohort likely represents a more clinically selected rhythm‐control population, which may partly explain the lower 30‐day readmission rate observed in this study [4, 14]. This difference is consistent with the selection of patients with greater clinical stability, planned rhythm‐control intervention, and structured peri‐discharge management. Among patients who were readmitted, atrial arrhythmia diagnoses remained the most common principal reasons for readmission, indicating that recurrent arrhythmia continues to be an important driver of early return to care even within a rhythm‐control strategy [14]. When compared with other procedure‐selected rhythm populations, however, the overall readmission rate in our cohort is similar to rates reported in published CA cohorts, which may reflect overlapping patient selection and peri‐discharge care pathways in rhythm‐control populations [15, 16, 17]. For context, analyses of the 2010–2014 NRD dataset reported 30‐day readmission rates of 14.4% after index AF hospitalization and 10.9% after CA, respectively [4, 15]. By contrast, Munir et al. [14] studied 388 340 AF hospitalizations based on the 2013 NRD dataset, which corresponds to an ICD‐9‐identified cohort, and found a significantly elevated readmission rate of 15.1% which was primarily due to repeat AF, HF exacerbation, and a high comorbid disease burden. In more recent analyses focusing on selected procedures, readmission rates have been found to be decreasing. For instance, Garg et al. [16] studied the 2014 NRD and reported a 30‐day readmission rate of 11% after CA, with nearly half of them occurring within 9 days post‐discharge and predominantly due to recurrent arrhythmia or HF. In a similar study, Pasupula et al. [17] analyzed NRD from 2016 to 2019 and found a similar rate of early readmission of 10.7% following combined left atrial appendage occlusion (LAAO) and AF ablation attributed chiefly to cardiac causes. Among those readmitted, the most common primary diagnosis was HF in the LAAO and CA cohort, whereas paroxysmal AF was most common in the CA‐only patients [17]. These studies highlighted care features associated with lower readmissions, which included same‐day ablation, treatment at high‐volume centers, and structured discharge with early clinic follow‐up ≤ 14 days after AF admission [14, 16]. The lower readmission rate in our study also likely reflects two contemporary practice changes: the broader use of direct oral anticoagulant (DOAC)‐based peri‐ and post‐cardioversion anticoagulation, which has resulted in low 30‐day events [18] and the adoption of “wait‐and‐see” pathways in the emergency department, with selective or delayed cardioversion and planned early follow‐up [19]. When combined with organized peri‐discharge care and evidence‐based pharmacotherapy, early recurrence and readmission are less likely. In addition, the comparatively lower readmission rate in this cohort may reflect broader evolution in HF and AF management and structured post‐discharge care pathways rather than the effect of any single therapy. However, because the NRD does not capture medication‐level data, including anticoagulant selection, antiarrhythmic therapy, HF guideline‐directed medical therapy, dosing, or adherence, medication‐based mechanistic explanations cannot be tested in this dataset and should be considered hypothesis‐generating. The absence of these variables also limits causal interpretation by introducing potential residual confounding and selection bias. Patients with prompt outpatient follow‐up, reliable access to anticoagulants and antiarrhythmic drugs (AADs), better medication adherence, optimized HF therapy, and sustained sinus rhythm after EC may have lower readmission risk through mechanisms not measured in the NRD [3, 20, 21, 22]. Conversely, patients with recurrent AF after discharge, suboptimal anticoagulation, medication intolerance, poor adherence, or limited outpatient access may be more likely to be rehospitalized, but these pathways cannot be separated from measured factors such as payer status, income quartile, comorbidity burden, and discharge disposition. Selection for inpatient EC may also be influenced by clinical stability, symptom burden, AF duration, anticoagulation eligibility, prior rhythm‐control history, and feasibility of outpatient follow‐up, none of which are fully captured by administrative codes. Therefore, the comparatively lower 30‐day readmission rate observed in this EC cohort should be interpreted as an association within a selected hospitalized rhythm‐control population, rather than as evidence of a causal effect of EC, medication strategy, anticoagulation quality, or post‐discharge care pathways on readmission.

In our study, readmitted patients incurred $4680 higher index‐stay charges (median $31 576 vs. $26 896; *p* < 0.001) and an additional $27 994 at readmission, underscoring a markedly higher economic burden. Despite the secular decline in readmissions described above, the pattern of increased financial burden among readmitted patients seen in our study aligns with earlier analyses of AF readmission [4, 23]. Increased healthcare utilization among readmitted patients likely stems from multiple factors like AF recurrence, which is a common and urgent cause of post‐cardioversion readmission; exacerbation of prevalent comorbidities such as COPD, diabetes, and HF, which can destabilize high‐risk patients [23, 24]; and gaps in transitional care, particularly among patients discharged to nonhome settings, which have been linked to adverse outcomes in prior studies [15]. Thus, even as overall readmission rates decline, the economic footprint of readmissions remains substantial, a signal that also aligns with worse clinical consequences observed during readmission. We reported four times higher in‐hospital mortality during readmission compared with the index hospitalization. This is consistent with prior US‐based evidence on outcomes after EC. In a large single‐center study of 1017 patients, although immediate procedural mortality was rare, 11% were readmitted and 1.4% died within 30 days, with adverse outcomes driven largely by comorbidities and clinical instability [25]. Jain et al. demonstrated differences in outcomes based on cardioversion success. Among patients with unsuccessful inpatient cardioversion, 83.3% were readmitted within 30 days and 33.3% died, whereas among those who underwent successful cardioversion 8.9% had 30‐day readmission and no deaths reported [26]. This contrast underscores that while comorbidity burden and hemodynamic vulnerability strongly predict adverse outcomes, procedural success itself is also a decisive determinant of post‐cardioversion prognosis. Our results underscore the vulnerability of the post‐discharge period following cardioversion and align with prior studies indicating that readmissions, rather than the index hospitalization, account for the majority of mortality risk.

In our multivariable analysis, comorbid COPD, HF, and diabetes emerged as the strongest clinical predictors of 30‐day readmission. This pattern is concordant with previous NRD‐based studies evaluating readmissions post‐ablation for AF. COPD, coronary artery disease (CAD), acute renal failure, and fluid/electrolyte disorders have been previously identified as independent predictors of readmission [16]. Similarly, Arora et al. [15] reported higher readmission risk with diabetes, HF, COPD, CAD, chronic kidney disease (CKD), prior stroke/transient Ischemic Attack (TIA), female sex, longer index LOS, and nonhome discharge. Together, these data corroborate our finding that cardiopulmonary comorbidity and broader multisystem disease burden drive early rehospitalization after rhythm‐control care. The association between these comorbidities and early rehospitalization after EC is biologically plausible. In COPD, hypoxemia, airway infection/inflammation, β‐agonist exposure, and autonomic imbalance promote atrial arrhythmogenesis, and COPD has been linked to poorer cardioversion durability mechanisms consistent with higher post‐EC recurrence and rehospitalization [27]. Lu et al. [28] in 2025 further demonstrated that COPD significantly elevated recurrence risk after ablation (adjusted OR: 13.42) and was associated with earlier recurrence and greater left atrial enlargement. In HF, neurohormonal activation of renin–angiotensin–aldosterone system (RAAS), elevated filling pressures, and left‐atrial stretch/fibrosis create a substrate that sustains AF and predisposes to relapse after EC [29]. Finally, diabetes contributes via structural (fibrosis/dilatation), electrical, and autonomic remodeling, with glycemic variability further increasing AF vulnerability. Diabetes has also been associated with lower cardioversion success and earlier AF recurrence [30]. Collectively, these studies support our findings, highlighting the central role of cardiopulmonary comorbidities in leading to adverse outcomes in AF.

We also found that female sex and older age were associated with a higher risk of readmission. These findings are consistent with prior studies showing that women with AF often report a greater symptom burden and lower quality of life, yet are less likely to receive rhythm control strategies [31, 32, 33]. Although acute success rates of EC are similar between sexes [28, 34], some studies report higher recurrence rates in women [35], potentially due to differences in atrial remodeling and hormonal influences. Although older adults often achieve immediate rhythm control, they may experience reduced long‐term rhythm maintenance due to age‐related atrial remodeling, particularly an increase in left‐atrial volume as a proportion of body surface area, which is an early predictor of recurrence following EC, with similar age effects on substrate also noted following CA and higher comorbidity prevalence [36, 37], In addition, insurance status is connected to the early reuse of care. An NRD study analyzing hospitalizations from 2010 to 2014 found that privately insured patients were less likely to readmit at 30 days compared to Medicare beneficiaries, and that the coverage of public insurance, which could be an indicator of advanced age, disability, or socioeconomic disadvantage, was independently associated with a greater risk of readmission following AF ablation [15]. Previous studies have shown that such populations face reduced follow‐up care [20], lower medication adherence due to cost [21], and more fragmented healthcare [38], highlighting the role of social determinants in post‐discharge outcomes.

Hospital‐level structural characteristics were also associated with 30‐day readmission. Compared with small hospitals, medium‐ and large‐bed‐size hospitals had higher adjusted odds of readmission, whereas nonmetropolitan hospital location was associated with lower adjusted odds. In HCUP data, hospital bed size represents the number of short‐term acute care beds set up and staffed, with categories defined according to hospital region, urban–rural designation, and teaching status; it is not a direct measure of procedural volume, electrophysiology expertise, quality of care, or patient severity [39]. Similarly, hospital location and teaching status are hospital‐level classifications, with hospitals categorized as metropolitan nonteaching, metropolitan teaching, or nonmetropolitan; this variable reflects the index hospital setting rather than the patient's residential geography, travel distance, outpatient access, or post‐discharge support [40]. The higher readmission odds in medium‐ and large‐bed‐size hospitals may reflect residual case mix, referral of more complex patients to larger hospitals, greater availability of specialized cardiovascular services, or unmeasured differences in discharge planning and post‐acute care pathways. Conversely, the lower odds observed for nonmetropolitan hospitals may reflect selective referral or transfer of higher acuity patients to metropolitan centers, differences in admission or readmission thresholds, geographic barriers to rehospitalization, or incomplete capture of out‐of‐state readmissions and out‐of‐hospital mortality. Prior readmission research indicates that hospital population case mix, institution‐specific factors, and hospital‐location patterns can influence 30‐day readmission estimates even after adjustment for measured patient characteristics [41, 42, 43]. Therefore, these hospital‐level associations should be interpreted as markers of hospital context, healthcare‐utilization patterns, and unmeasured clinical complexity rather than causal effects of hospital size or nonmetropolitan care.

Conversely, hypertension, obesity, higher socioeconomic status, and cardiac arrest were inversely associated with 30‐day readmission. In our cohort, hypertension and obesity were less frequent among readmitted patients in descriptive analyses and remained inversely associated with readmission after multivariable adjustment; however, the NRD does not contain blood pressure values, body mass index (BMI) values, obesity severity, outpatient medication use, medication adherence, weight‐management data, or longitudinal measures of hypertension or obesity control. Therefore, whether hypertension or obesity were more consistently controlled in these patients could not be directly assessed. For obesity, the observed association may be partly consistent with reports of better short‐term cardiovascular outcomes among patients with established cardiovascular disease and AF, but it may also reflect residual confounding, differences in treatment intensity, BMI coding, reverse causation, or variation in anticoagulation management among obese patients receiving rhythm‐control care [44, 45, 46, 47]. Similarly, the inverse association for hypertension may reflect greater health system contact, more frequent use of cardiovascular therapies, or unmeasured outpatient disease control rather than a protective effect of hypertension itself as hypertension remains an established risk factor and common comorbidity in AF [48].

The inverse association for cardiac arrest requires particular caution because 30‐day readmission is conditional on survival to discharge and eligibility for subsequent rehospitalization. Patients with cardiac arrest during the index hospitalization may have higher early mortality, require longer inpatient stabilization, undergo transfer to higher‐acuity care, or follow different discharge pathways, which may reduce the observed probability of rehospitalization. Therefore, this association may reflect survival‐to‐discharge conditioning and competing risk from index hospitalization death rather than lower post‐discharge vulnerability [9]. In addition, because this analysis relies on administrative diagnosis and procedure codes, differences in documentation practices and the use of secondary diagnosis codes may influence estimates for complications and comorbidities [10, 13]. Higher socioeconomic status may be associated with lower readmission risk, although the NRD cannot determine the specific mechanism. Potential explanations include better access to outpatient follow‐up, lower cost‐related medication nonadherence, and less fragmented care [20, 21, 38]. Collectively, these inverse associations should be considered hypothesis‐generating and interpreted in the context of residual confounding, coding bias, and survivor bias rather than causal protection.

Emerging evidence supports the value of structured transitional care models (TCM) in reducing readmission risks. A recent US‐based meta‐analysis by Bilicki et al. [49] demonstrated that early outpatient follow‐up visits reduced 30‐day readmission rates in patients being discharged with HF by 27%. The same trend was observed with stroke, but no significant improvement was indicated with COPD [49]. At the policy level, Bindman et al. [22] found that Medicare beneficiaries receiving TCM services had significantly lower 30‐day mortality and healthcare costs. Collectively, these findings highlight the importance of both individualized clinical vigilance and systemic interventions to improve survival and reduce rehospitalization risk after EC.

### 4.1 Strengths and Limitations

This is the first study to analyze 30‐day readmission rates in AF patients following EC. The analysis benefits from the use of the NRD, which offers a large, all‐payer, nationally representative sample across diverse hospital settings in the US. The large cohort size and robust adjustment for a wide array of clinical and sociodemographic factors strengthen the generalizability and validity of the results. By focusing on patients specifically receiving EC treatment, the study provides targeted insights into the index hospitalization and readmission outcomes associated with this commonly employed rhythm‐control approach.

Our study has several limitations. The NRD does not include data on outpatient care, medication adherence, anticoagulation status, AAD use, HF guideline‐directed medical therapy, treatment changes after discharge, procedural success, or maintenance of sinus rhythm after EC. The NRD also does not reliably capture prior CA history, subsequent outpatient CA after discharge, or whether the index EC represented a first or repeat cardioversion, because prior procedural history codes are nonspecific and outpatient procedures are not included in the database. Because prior or subsequent ablation and prior cardioversion history may influence rhythm‐management trajectory, selection for inpatient EC, and the likelihood of 30‐day readmission, residual confounding may persist despite multivariable adjustment. Similarly, differences in follow‐up access, medication use, treatment adherence, anticoagulation quality, and rhythm recurrence could not be directly assessed as contributors to readmission risk. AF subtype may influence post‐EC outcomes, but AF subtype‐specific analyses were not performed because this study was designed to evaluate overall readmission risk after inpatient EC, and administrative coding does not reliably capture AF duration, rhythm burden, prior rhythm‐control history, outpatient recurrence, or sustained sinus rhythm after discharge with sufficient clinical granularity. Although the model adjusted for hospital location and teaching status, the NRD does not provide individual‐level household income, family size, residential geography, distance to care, or outpatient access measures. Therefore, residual confounding by individual socioeconomic status and geography may persist despite adjustment. Finally, because this analysis reflects practice patterns during 2016–2017, subsequent changes in AF management and cardiometabolic therapy may influence contemporary readmission risk and should be evaluated in future studies using more recent data sources with medication linkage. The inability to track out‐of‐state readmissions or out‐of‐hospital mortality may lead to an underestimation of events. Additionally, the use of administrative codes may introduce misclassification, and the observational nature of the study prevents definitive conclusions regarding causality. Although missing data were limited to 1.49% of the unweighted analytic sample and complete versus incomplete cases were generally balanced across outcomes and comorbidities, multiple imputation was not performed. Despite these constraints, the results offer valuable insights into potentially modifiable factors that influence readmission risk following EC.

## 5 Conclusion

In this nationally representative cohort of patients hospitalized for AF undergoing EC, the 30‐day all‐cause readmission rate was approximately 1 in 10. Readmissions were accompanied by higher in‐hospital mortality and a substantially greater economic burden, with $4680 higher index‐stay charges and an additional $27 994 at readmission, reflecting prolonged hospital stays, increased healthcare utilization, and post‐discharge instability. Among clinical and sociodemographic factors, COPD, diabetes, HF, liver disease, peripheral vascular disease, and Medicaid coverage emerged as the strongest independent predictors together with older age and female sex. These findings highlight the need for comprehensive post‐discharge care that addresses comorbidities alongside rhythm management to prevent early readmissions and reduce their healthcare burden.

## Author Contributions

**Adeena Jamil:** conceptualization, methodology, data curation, formal analysis, writing – original draft, visualization, and project administration; **Muhammad Umer Sohail:** conceptualization, methodology, data curation, formal analysis, writing – original draft, validation, and writing – review and editing. **Asad Ali Ahmed Cheema:** conceptualization, methodology, project administration, supervision, writing – original draft, and writing – review and editing; **Syed Usama Ashraf:** data curation, investigation, resources, writing – review and editing, and visualization. **Anushah Faheem Ilyas:** data curation, investigation, resources. **Faizan Abbas:** data curation, validation, writing – review and editing, and supervision. **Shaikh Jehanzaib Saeed:** investigation and data curation. **Mohammad Rayyan Faisal:** investigation and data curation. **Ahmad Murtaza Anwar:** investigation and writing – review and editing. **Shanzey Rai:** data curation and visualization. **Syeda Unzila Tirmizi:** data curation. **Muhammad Uzair:** investigation. **Tooba Shahzad:** data curation and visualization.

## Funding

The authors have nothing to report.

## Disclosure

This manuscript is original, has not been published previously, and is not under consideration elsewhere. Declaration of Generative AI in Scientific Writing: No generative AI or AI‐assisted tools were used in the preparation of this manuscript.

## Conflicts of Interest

The authors declare no conflicts of interest.

## Data Availability

This study is based on the publicly available Nationwide Readmissions Database (NRD), a Healthcare Cost and Utilization Project (HCUP) database sponsored by the Agency for Healthcare Research and Quality (AHRQ). Information on database access and purchase is available at: https://hcup‐us.ahrq.gov/nrdoverview.jsp.
